# Angle-resolved photoemission spectroscopy of liquid water at 29.5 eV

**DOI:** 10.1063/1.4979857

**Published:** 2017-04-07

**Authors:** Junichi Nishitani, Christopher W. West, Toshinori Suzuki

**Affiliations:** Department of Chemistry, Graduate School of Science, Kyoto University, Kitashirakawa Oiwake-cho, Sakyo-Ku, Kyoto 606-8502, Japan

## Abstract

Angle-resolved photoemission spectroscopy of liquid water was performed using extreme ultraviolet radiation at 29.5 eV and a time-of-flight photoelectron spectrometer. SiC/Mg coated mirrors were employed to select the single-order 19th harmonic from laser high harmonics, which provided a constant photon flux for different laser polarizations. The instrument was tested by measuring photoemission anisotropy for rare gases and water molecules and applied to a microjet of an aqueous NaI solution. The solute concentration was adjusted to eliminate an electric field gradient around the microjet. The observed photoelectron spectra were analyzed considering contributions from liquid water, water vapor, and an isotropic background. The anisotropy parameters of the valence bands (1*b*_1_, 3*a*_1_, and 1*b*_2_) of liquid water are considerably smaller than those of gaseous water, which is primarily attributed to electron scattering in liquid water.

## I. INTRODUCTION

Ultrafast spectroscopy using femtosecond lasers has revolutionized the studies of chemical reaction dynamics in atomic time- and length-scales to open a new research area of *femtochemistry*.^1^ Besides many other achievements, real-time observation of non-adiabatic dissociation of NaI by Zewail and coworkers is the landmark in this field.^2^ As represented by this example, photochemical reactions ubiquitously involve non-adiabatic transitions among potential energy surfaces and branching into different reaction products. For elucidating such complex reaction dynamics, it is crucial to observe the entire wave packet motion from the Franck-Condon region to the final products. Time-resolved photoelectron spectroscopy (TRPES) enables such experiments,^3^ if a sufficiently short probe wavelength becomes available to induce ionization from all electronic states of the reactant, transient species, and the products. Such a pulsed light source should be operated at a high repetition rate (on the order of kHz to MHz) to accumulate the experimental data while limiting the number of photoelectrons generated per laser shot to avoid space charge effects. High harmonic generation (HHG) driven by a high repetition-rate laser is a promising tool for TRPES from this point of view.^4–9^

HHG is induced by focusing a driving laser beam (typically 800 nm and 1 mJ/pulse) into rare gas.^10–14^ It creates a number of odd harmonics simultaneously,^15^ supporting an attosecond pulse (or train) in the time domain. However, photoelectron spectroscopy (PES) in the frequency domain requires an adequate energy resolution, so that the photon energy distribution should be narrowed by sacrificing the pulse duration.^4–6,8,9,16,17^ For this purpose, time-preserving or time-delay compensated monochromators^18,19^ are usually employed. These monochromators have excellent performances, while their designs are complex and care must be taken to maintain a constant photon flux and directional pointing of monochromatized radiation when changing the laser polarization. Therefore, the present study employs SiC/Mg coated mirrors to select the 19th single-order harmonic to ensure a constant photon flux for different polarizations.

Photoelectron spectroscopy (PES) has been widely applied to solid and gas targets, while liquids have been scarcely studied with this method, because PES requires high vacuum conditions. Nonetheless, Delahay, Siegbahn, and their coworkers have, respectively, challenged ultraviolet (UV) and X-ray PES of liquids since the late 60s.^20,21^ Faubel introduced a liquid microjet technique in the late 80s, which has reduced the technical difficulty associated with the introduction of volatile liquids into high-vacuum.^22^ Since the late 90s, a series of soft X-ray PES studies have been performed by Winter, Faubel, and their coworkers using synchrotron radiation.^23^ More recently, TRPES of liquids has started in 2010 for real-time study of solution chemistry,^24–26^ in which time and angle-resolved photoelectron spectroscopy (TARPES) has been demonstrated using femtosecond UV lasers.^27,28^ TRPES using extreme UV (EUV) and near infrared (800 nm) femtosecond (or attosecond) lasers has also been reported,^4–9,29^ while the only example of angle-resolved PES (ARPES) of liquid in the EUV range is by Abel and coworkers at 38.7 eV.^9,30^ Photoionization of liquid water is an important target for ARPES from the viewpoint of radiation chemistry and biology, because ionization of cell water, which constitutes more than 70% of the living cell, is the initial step of radiation damages to a cell induced by high-energy particles or electromagnetic radiation. As an initial step of our effort toward EUV-TARPES, we present here ARPES of liquid water using our EUV light source at 29.5 eV.

## II. EXPERIMENTAL

**FIG. 1.**
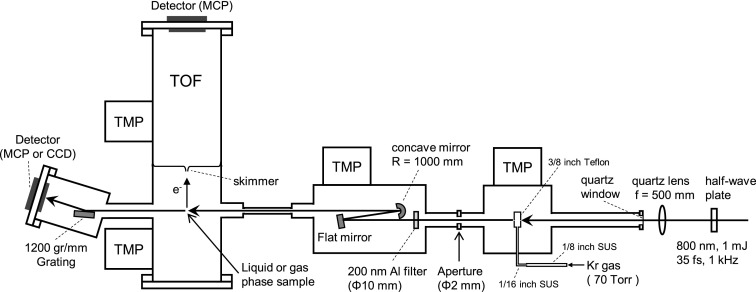
Schematic diagram of our apparatus.

A schematic diagram of our experimental apparatus is shown in Fig. 1. A part of the output (1 mJ) from a one-box Ti-sapphire regenerative amplifier (Coherent Astrella, 35 fs, 800 nm, FWHM 40 nm, 1 kHz, 6 mJ) is focused using a quartz lens (f = 500 mm) into Kr gas filled in a 3/8 in. Teflon tube with 6.35 mm inner diameter. The pressure in this tube could not be measured, while the Kr pressure in a 1/8 in. SUS tube connected to the Teflon tube was measured to be 70 Torr. The driving laser beam passes across the Teflon tube through two pinholes drilled by the laser beam itself. The beam waist of the driving laser in the tube was estimated to be 0.2 mm. The Teflon tube is in a vacuum chamber, and Kr gas leaking through the pinholes is pumped out by a turbo molecular pump (TMP). The high harmonics generated in the Kr gas and the remaining driving laser beam co-propagate and enter the optics chamber, in which the driving laser beam is attenuated by an Al filter (200 nm thickness) and the 19th order single-ordered harmonic is selected using a concave (R = 1000 mm) and a flat SiC/Mg mirror, which are specifically designed to selectively reflect the 19th harmonic. The diameter of EUV light at the sample was measured to be 40 *μ*m in FWHM using photoionization of the microjet of 25 *μ*m in diameter. The photon flux was estimated to be 2 × 10^6^ photon/pulse at maximum using a calibrated EUV photodetector (Opto Diode Corp., AXUV100Al).

A liquid sample is pressurized using an HPLC gradient flow pump and degassed using a built-in degasser. The sample liquid is transferred from the degasser to a liquid-discharging nozzle using a temperature-controlled PEEK tube. The liquid is discharged into a photoelectron spectrometer through a fused silica capillary of 7 mm in length and 25 *μ*m in inner diameter. The EUV laser beam crosses with the liquid microjet at 1 mm downstream from the capillary nozzle. Photoelectrons emitted from the microjet are sampled into a linear time-of-flight (TOF) analyzer using a 1-mm diameter Al entrance skimmer, coated with graphite, placed at 2 mm from the ionization point. The photoelectron spectrometer has been designed for X-ray PES, and it has two fine wire meshes after the entrance skimmer to decelerate incoming electrons. These meshes were electrically grounded in the experiments described below; however, they created weak background signals due to scattering. The detection solid angle is about 1 msr, determined by the radius (38 mm) of a microchannel plate detector and the flight distance (1200 mm). The electron signal from the microchannel plate is preamplified and counted using a multichannel scaler. The typical pressures in the photoionization chamber and the TOF analyzer are 1 × 10^−4^ and 1 × 10^−6 ^Torr, respectively.

We examined the spectrum of the monochromatized EUV light in two ways: one is one-photon ionization of rare gases and the other is direct spectroscopic measurement of EUV light using a home-made EUV spectrometer. These measurements indicated that the intensity of the adjacent, the 17th and 21st, harmonics was less than 4% of the 19th, in agreement with the expected performance of the SiC/Mg mirrors. The linear polarization of the 19th harmonic is rotated using a half-wave plate for the driving laser (800 nm).^31,32^ Variation of the EUV photon flux was less than 4% when rotating polarization.

**FIG. 2.**
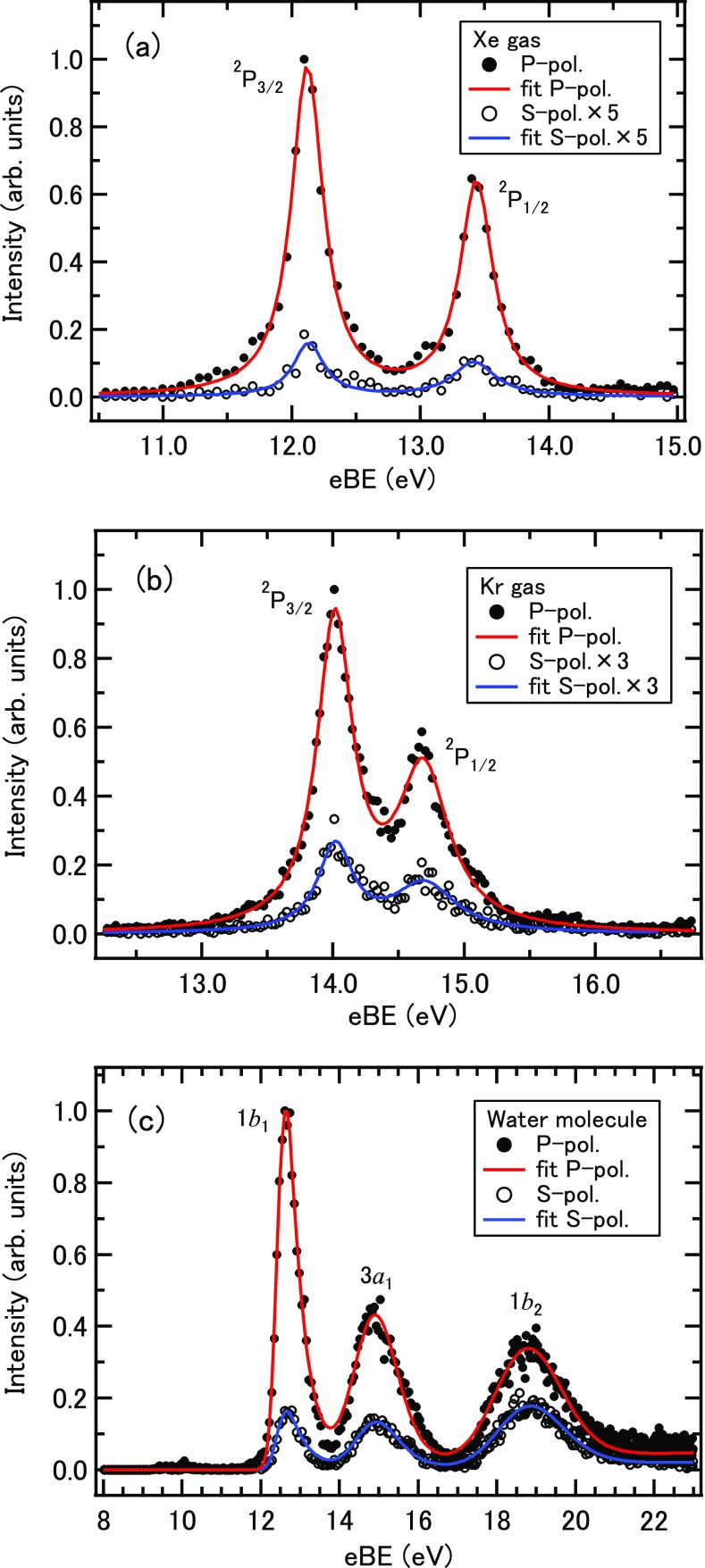
Photoelectron spectra of (a) Xe, (b) Kr, and (c) water molecules measured using P and S polarizations of EUV light.

Figures 2(a) and 2(b), respectively, show the photoelectron spectra of Xe and Kr measured using P and S polarizations, in which P and S correspond to the linear polarization parallel and perpendicular to the electron detection axis. The lines observed in these spectra are considerably broader than their intrinsic linewidth previously measured by high-resolution photoemission spectroscopy, and the width (0.5 eV in FWHM) is primarily ascribed to the spectrum of EUV radiation. (The spectrum of EUV radiation varies with the Kr pressure and the driving laser intensity.) The fine structure splitting (0.665 eV) of Kr^+^ is not well resolved, while that of Xe^+^ (1.306 eV) is resolved with our resolution. The line shape is expressed by a Lorentzian. Figure 2(c) shows the photoelectron spectra of water molecules and the least squares fitting using exponentially modified Gaussians (EMGs); the functional form of EMG is described later. Three valence bands of X(^2^B_1_), A(^2^A_1_), and B(^2^B_2_) are clearly seen. The vibrational structures of water bands are not well resolved owing to the large spectral width of our EUV light: however, the Franck-Condon envelopes agree with the literature. A small band at an electron binding energy (eBE) of 10 eV is the 1*b*_1_ band caused by the 21st harmonic; the observed spectra are shown against eBE calculated for the 19th harmonic, so that photoelectron bands caused by other harmonics are horizontally shifted by 3.1 eV. The weak continuous background signal seen in the water spectrum is due to electron scattering at the wire meshes described in Section II.

The photoemission anisotropy in a one-photon process is expressed by the following equation:

$$I\left(\theta \right)\propto 1+\beta_{2}P_{2}(\text{cos}\;\theta ),$$ (1)

where θ is the relative angle between the laser polarization and the electron detection angle and $P_{2}(x)$ is the second-order Legendre polynomial. $\beta_{2}$ is called an anisotropy parameter and is determined by the equation

$$\beta_{2}=\frac{I\left(0\right)-I(90)}{\left(\frac{I\left(0\right)}{2}\right)+I(90)},$$ (2)

where I(0) and I(90) are the intensities of the photoelectron bands measured using P and S polarizations, respectively. Table I lists the determined anisotropy parameters and literature values.

**TABLE I.** Photoemission anisotropy parameters $\left(\beta_{2}\right)$ of rare gases and gaseous and liquid water.

	Photon energy (eV)	^2^*P*_1/2_	^2^*P*_3/2_		
Xe^+^	26.86	1.86 ± 0.05	1.98 ± 0.04		Dehmer *et al.*^33^
Xe^+^	40.81	1.58 ± 0.03	1.28 ± 0.03		Dehmer *et al.*^33^
Xe^+^	29.5	1.82 ± 0.03	1.83 ± 0.03		This work
Kr^+^	26.86	1.50 ± 0.06	1.60 ± 0.05		Dehmer *et al.*^33^
Kr^+^	40.81	1.93 ± 0.06	1.90 ± 0.07		Dehmer *et al.*^33^
Kr^+^	29.5	1.49 ± 0.02	1.53 ± 0.02		This work
		1*b*_1_	3*a*_1_	1*b*_2_	
Gas water	30.0	1.31	0.93	0.49	Banna *et al.*^34^
Gas water	29.5	1.21 ± 0.05	0.86 ± 0.05	0.44 ± 0.06	This work
Gas water (Liquid)^a^	29.5	1.16 ± 0.06	0.79 ± 0.07^b^	0.38 ± 0.07	This work
Liquid water	29.5	0.27 ± 0.07	0.24 ± 0.09	0.18 ± 0.06	This work

^a^ The row of “Gas water (Liquid)” indicates anisotropy parameters of gaseous water extracted from the liquid water spectrum.

^b^ As we assume in our analysis that both peaks in the 3*a*_1_ doublet are identical, we present a single anisotropy parameter for this peak.

## III. RESULTS AND DISCUSSION

**FIG. 3.**
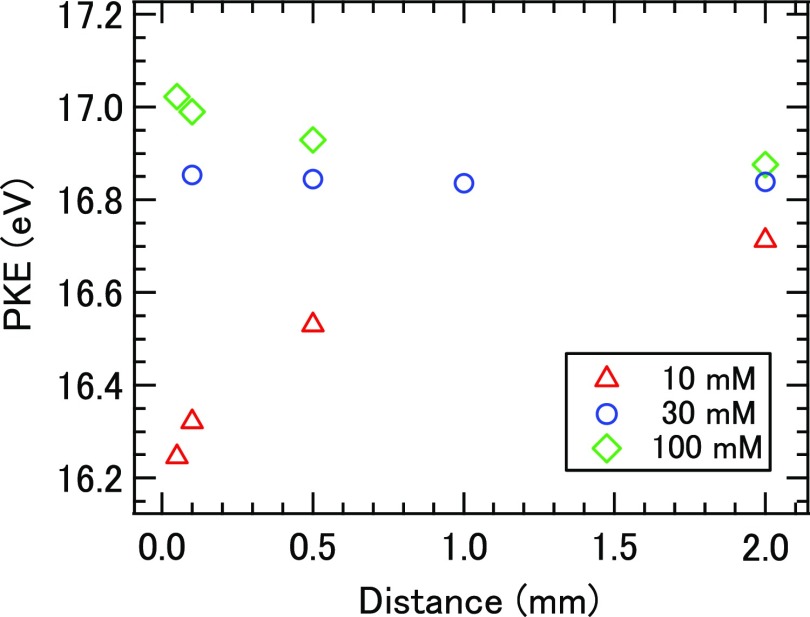
Distance dependence of PKE measured for the 1*b*_1_ band of water molecules.

Previously, Kurahashi *et al.* have shown that the surface potential of a liquid microjet is minimized at the NaX (X = halogen) concentration around 30 mM at the flow rate of 0.5 ml/min.^35^ In the present study, we employed the same design of a liquid-discharging nozzle assembly, for which we confirmed, using completely different instruments, the same behavior of the surface potential as reported by Kurahashi *et al.*; they employed synchrotron radiation and a hemispherical electron energy analyzer, while we used laser high harmonics and a linear TOF analyzer. Figure 3 shows the photoelectron kinetic energy (PKE) associated with the 1*b*_1_ band of water molecules measured as a function of the distance of the ionization point from the liquid microjet. One can see that PKE is independent of the distance from the liquid microjet at the concentration of 30 mM, which implies that the electric potential of a liquid surface is equal to that of the entrance skimmer of the spectrometer. On the other hand, when using aqueous 10 and 100 mM solutions, PKE varies with the distance in opposite ways to each other. Thus, in order to avoid any influence of the electric field to photoemission anisotropy, we have employed the 30 mM solution in most of our experiments, while we used other concentrations for the purpose of comparison.

**FIG. 4.**
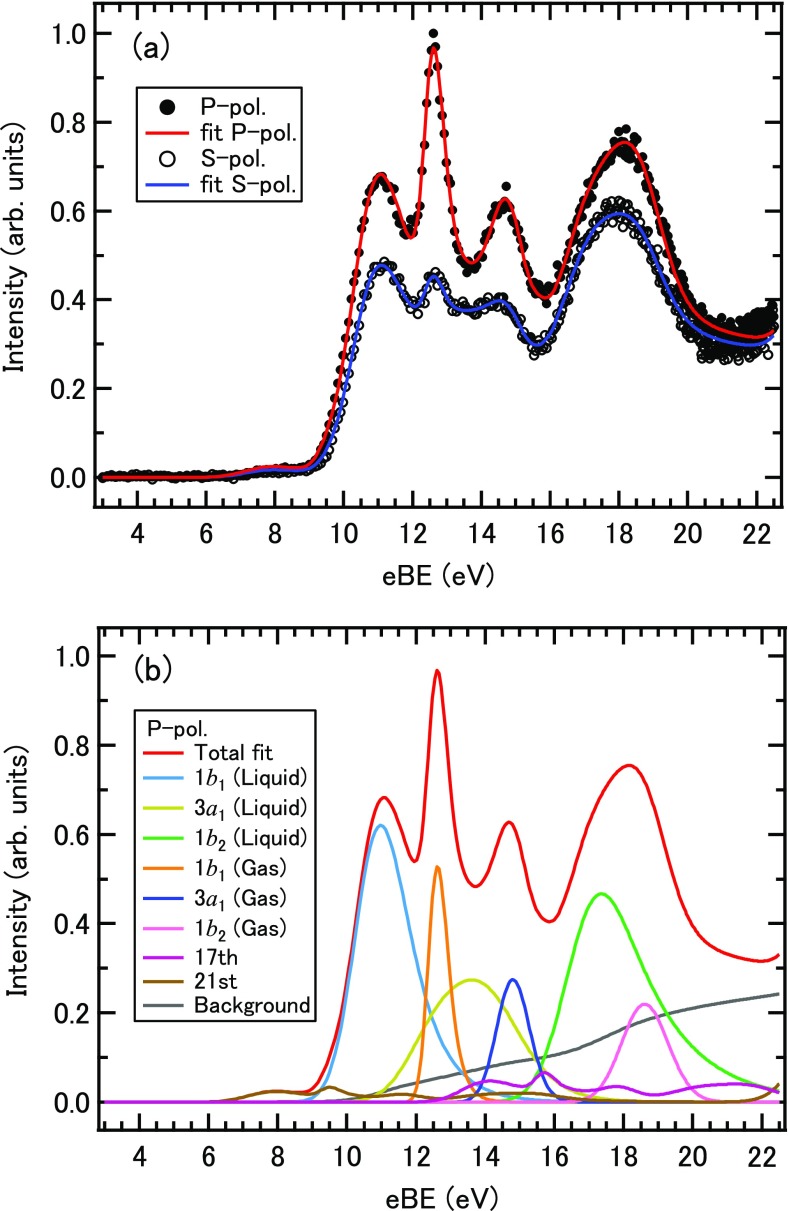
(a) Photoelectron spectra measured for the 30 mM solution using P and S polarizations. (b) Each component obtained by the least squares fitting for the photoelectron spectrum measured using P polarization.

Figure 4(a) shows the photoelectron spectrum measured for the 30 mM solution using P and S polarizations. A background signal growing towards high eBE (low PKE) arises mainly from inelastic scattering of photoelectrons in solution, while some contributions of the secondary electrons generated by electron impact ionization and intermolecular Coulombic decay^36,37^ are also expected to contribute in the high eBE region. The valence bands appearing in the low eBE region are of liquid water and also water vapor evaporated from the jet surface. We performed least squares fitting of the spectra by assuming contributions from the liquid, vapor, and a broad background from inelastic scattering in water. The former two were expressed as a sum of EMGs

$$S_{Bands}(E;\mu_{i},\sigma_{i},\lambda_{i})=\sum_{i}A_{i}\left(\frac{\lambda_{i}}{2}e^{\frac{\lambda_{i}}{2}\left(2(\mu_{i}+C_{S})+\lambda_{i}\sigma_{i}^{2}-2E\right)}\;erfc\left(\frac{(\mu_{i}+C_{S})+\lambda_{i}\sigma_{i}^{2}-E}{\sigma_{i}\sqrt{2}}\right)\right),$$ (3)

where $S_{Bands}$ is the contribution to the fit from the photoelectron bands, *E* is the electron binding energy, *i* is the number of EMGs included in the fit, and $A_{i}$ is the amplitude of the function. The parameters $\mu_{i}$, $\sigma_{i}$ and $\lambda_{i}$ are the mean and variance of the Gaussian and the rate of the exponential, respectively. The values of $\mu_{i}$ for the 1*b*_1_, 3*a*_1_, and 1*b*_2_ bands were set to literature values. Finally, $C_{S}$ is a constant to account for the PKE shift caused by the liquid surface potential, considered independently for both gas and liquid water; however, we restricted the bands of the same species (gas or liquid) to take the same $C_{S}$ value to maintain the energy spacing of photoelectron bands. The relative intensities and energy separations of the vapor bands were fixed to the values determined for water molecules independently (Fig. 2(c)). However, the bandwidths were included in the fitting parameters, as these values show considerable variance in the vicinity of the liquid microjet (Fig. 6). The 3*a*_1_ band of liquid water was expressed using a doublet of identical EMGs with equal intensities, similar to the previous soft X-ray PES by Nishizawa *et al*.^38^ There were also small signals from the neighboring harmonic orders weakly reflected by our SiC/Mg mirrors. The contributions from the 17th and 21st harmonics were treated similar to the 19th harmonic and so described as

$$S_{OHO}\left(E\right)=C_{17th}S_{Bands}\left(E;\mu_{i}+3.1,\sigma_{i},\lambda_{i}\right)+C_{21st}S_{Bands}(E;\mu_{i}-3.1,\sigma_{i},\lambda_{i}),$$ (4)

where $C_{17th}$ and $C_{21st}$ are constant factors of the 17th and 21st harmonic functions, respectively. The broad background component was estimated from the liquid water spectrum using an empirical formula based on the method described in Ref. 39. Briefly, the model assumes that the background is created by inelastic scattering and that the energy-loss function is independent of photoelectron kinetic energy. The number of scattered electrons at a certain energy is calculated in proportion to the number of unscattered electrons observed at lower eBE in the spectrum

$$I_{Background}\left(E\right)\propto \int_{E^{\prime }=0}^{E}\;I_{Spectrum}\left(E^{\prime }\right)\;dE\prime ,$$ (5)

where $I_{Background}$ and $I_{Spectrum}$ are the intensities of the simulated background and the spectrum, respectively. It is noted here that if we simulate the background signal independently for P and S polarizations using Eq. (3), the anisotropy of the background becomes identical with that of unscattered electrons. This is unphysical, because the background electron signal generated by inelastic scattering in the liquid should exhibit lower anisotropy than the unscattered electron signals. Thus, we generated the common background signal function for P and S polarizations from the sum of liquid bands observed using these polarizations, which provided an entirely isotropic background signal

$$I_{Background}\left(E\right)=C_{BKG}\int_{E^{\prime }=0}^{E}\;(S_{P}^{i=Liquid}\left(E\prime \right)+S_{S}^{i=Liquid}(E\prime ))\;dE\prime ,$$ (6)

where $S_{P}^{i=Liquid}$ and $S_{S}^{i=Liquid}$ are the intensities of the liquid bands observed in the P and S spectra, respectively, and $C_{BKG}$ is a constant factor of the background function. Since the liquid water spectrum itself is determined by the least squares fitting, we performed iterative fitting to obtain consistent results between the estimated water spectrum and the broad background calculated from it. The photoelectron signals from Na^+^ and I^–^ were sufficiently small to neglect. The function for the fit is then:

$$F(E)=S_{Bands}\left(E\right)+S_{OHO}\left(E\right)+I_{Background}\left(E\right).$$ (7)

The spectra for P and S polarizations were fitted simultaneously and the parameters $\mu_{i}$, $\sigma_{i}$, and $\lambda_{i}$ for the photoelectron bands were maintained between spectra. Each component obtained by the least squares fitting for P polarization is indicated in Fig. 4(b). The anisotropy parameter *β*_2_ of each band was determined from the intensity ratio measured using P and S polarizations, as listed in Table I. The errors quoted with the anisotropy parameters were calculated from the experimental uncertainty derived from the energy resolution of the instrument and the variation in photon flux between P and S polarizations and the uncertainty in the fit. Owing to the surface reflection by the liquid microjet, the excitation probability is slightly different between P and S polarizations; however, its influence is negligible. Since the intensity due to inelastically scattered electrons continuously increases toward higher eBE (or lower PKE), ambiguity in the least squares fitting of the spectrum inevitably increases for the high eBE bands of 3*a*_1_ and 1*b*_2_ of liquid water. On the other hand, the 1*b*_1_ band of liquid water is almost isolated from other contributions, so that its *β*_2_ value is most reliable. The *β*_2_ value of the liquid 1*b*_1_ band is clearly smaller than the corresponding value of water molecules.

**FIG. 5.**
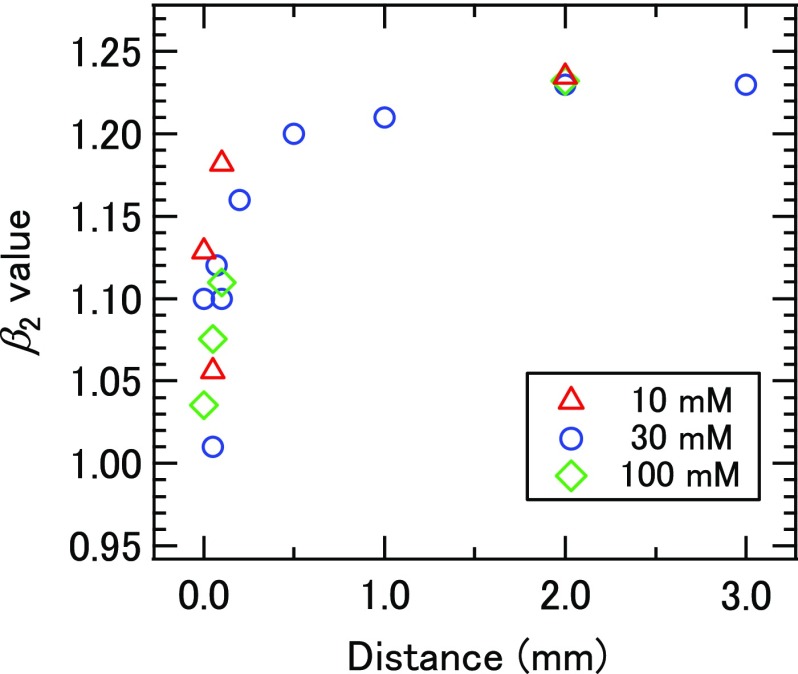
Distance dependence of photoemission anisotropy of the 1*b*_1_ band of water molecules.

The spectral analysis described above indicates that the anisotropy parameters extracted for gaseous species in the vicinity of a microjet are very similar to, yet slightly smaller than those of water molecules. Thus, we have measured the photoemission anisotropy of water molecules more closely as a function of the distance from the liquid jet surface as shown in Fig. 5. The result indicates that the anisotropy diminishes as the ionization point approaches the microjet. Since this occurs similarly for different NaI concentrations, the reduction of the anisotropy parameter is not ascribed to the liquid surface potential. There are at least three possible causes for the reduced photoemission anisotropy near the microjet. First, if too many photoelectrons are generated by an EUV laser pulse, the space charge reduces the anisotropy parameter. Secondly, if water clusters have high densities near the microjet, they would exhibit smaller anisotropy than water molecules. Finally, if electron-molecule scattering occurs in the evaporated gas, the anisotropy parameters diminish for all photoelectrons.

**FIG. 6.**
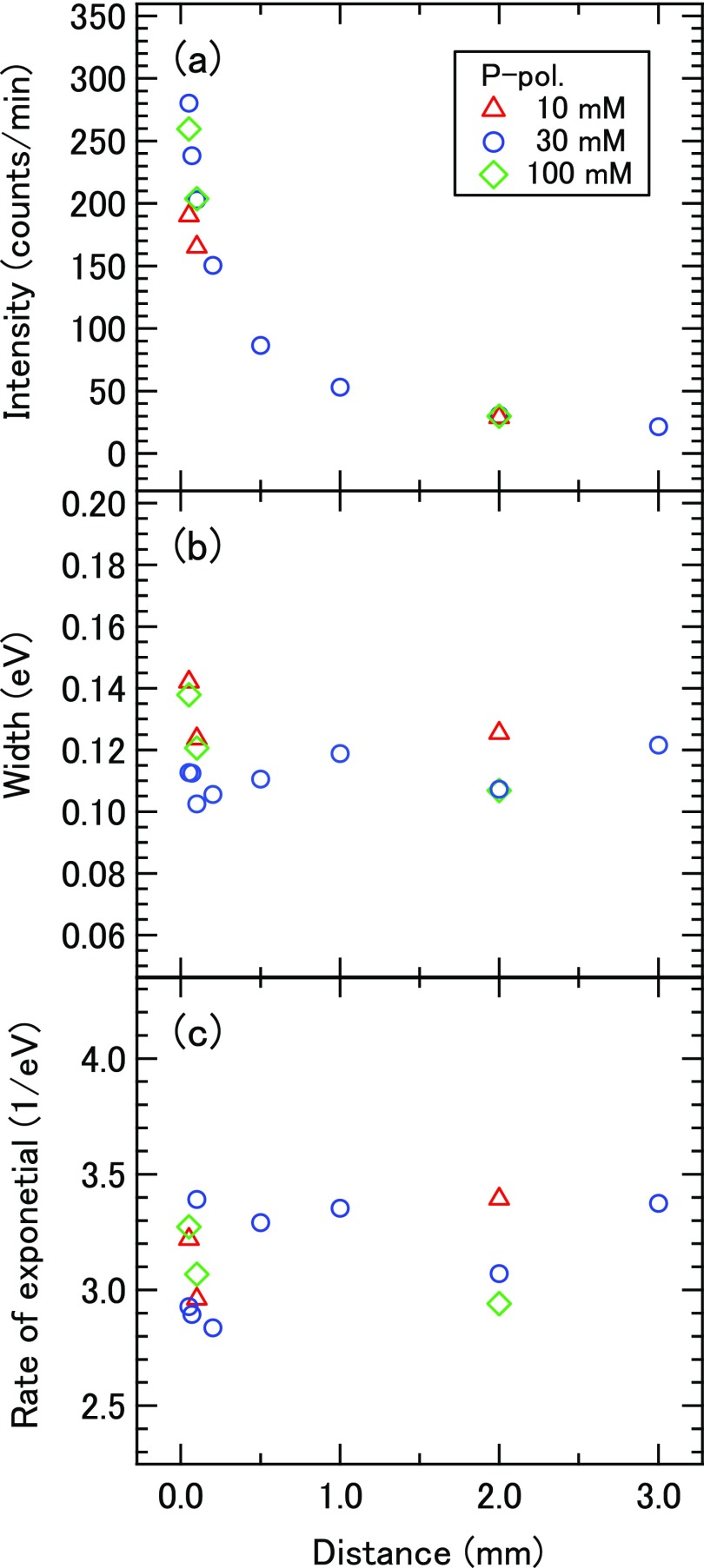
Distance dependence of (a) photoelectron intensity of the 1*b*_1_ band of water molecules, (b) the width of the fitted Gaussian, and (c) the rate of exponential of the EMG.

As for the first point, Figure 6 shows the observed photoelectron intensity of 1*b*_1_ band, the width of the fitted Gaussian, and the rate of exponential of the EMG as a function of the distance from the microjet. When the photoelectron intensity increases near the microjet, the width of the Gaussian and rate of exponential do change slightly. Although we examined the *β*_2_ values by changing the EUV laser intensity, the intensity could be attenuated only by a factor of three to maintain a reasonable signal to noise ratio; within this intensity range, the *β*_2_ values did not noticeably change. As for the second point, when a high-pressure liquid jet is discharged into vacuum, the internal pressure of the liquid abruptly diminishes and the interfacial wall is suddenly removed. This causes hydrodynamic instability, which might induce explosive evaporation of water molecules. Evaporated gas stagnates above the liquid surface and undergoes supersonic expansion, which lowers the gas pressure and possibly causes cluster formation. However, if cluster formation is the main cause for the reduction of anisotropy, it is not expected to depend so strongly on the distance. Finally, the momentum transfer cross-section between an electron and water molecule at the electron kinetic energy of 10 eV is about 8.5 × 10^−16^ cm^2^.^40^ If we assume that the water vapor pressure above the liquid is saturated (the number density of 2.3 × 10^17 ^cm^−3^ at 5 °C) and diminishes inversely proportional to the distance, the average number of collisions is estimated to be about 1. At the moment, the cause for the reduction of anisotropy near the jet surface is not totally clear. However, the space charge effect is the most likely cause. If this is the case, then the photoemission anisotropy parameters determined for both gaseous and liquid water by our experiment will in general be underestimated. For the 1*b*_1_ peak of gaseous water, which we might expect to show the largest reduction in the anisotropy parameter, this underestimation is less than 0.2. As the intrinsic anisotropy of the photoelectron feature decreases, the underestimation decreases proportionally.

**FIG. 7.**
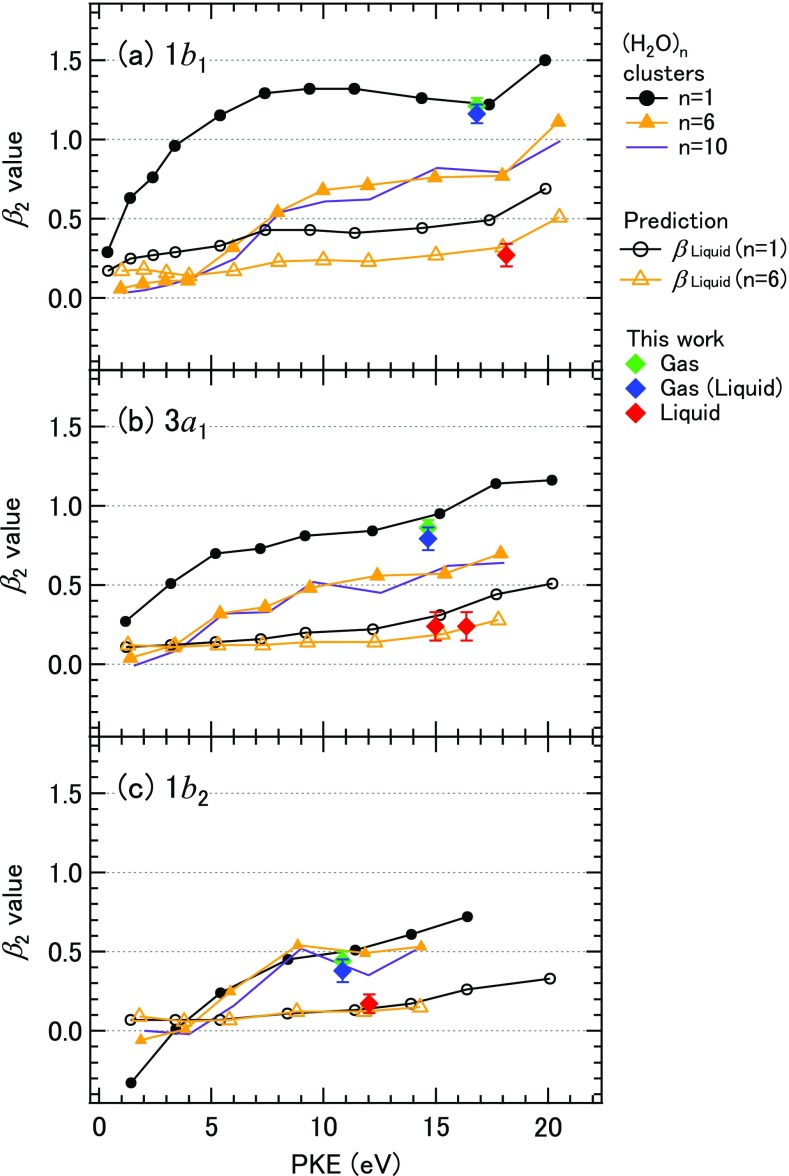
Comparison of anisotropy parameters determined in this work and those obtained by Hartweg *et al.*^43^ for (a) 1*b*_1_, (b) 3*a*_1_, and (c) 1*b*_2_ bands of water. Experimental values for H_2_O monomer and (H_2_O)_n_ clusters and predicted values for liquid water were taken from Ref. 43. Open black circles indicate the prediction based on the *β*_2_ values of monomer, and open orange triangles indicate the prediction based on cluster values (n = 6). The experimental *β*_2_ values determined for water molecules in this work are shown by green diamonds. Blue diamonds are *β*_2_ values determined for water molecules in the liquid water spectrum. The *β*_2_ values for liquid water are indicated by red diamonds. Two values are given for the liquid 3*a*_1_ band, as this band has been analyzed as a doublet.

Photoemission angular anisotropy provides valuable insights into an electronic character of the ionized state. Thürmer *et al.* have examined the photoemission angular distribution for soft X-ray O(1s) spectroscopy of liquid water using synchrotron radiation and demonstrated that the anisotropy is smaller than that of a water molecule.^41^ Hergenhahn and coworkers have measured photoemission anisotropy for neutral water clusters with the aggregation numbers (*n*) of 50–80 and attributed the diminished photoemission anisotropy to elastic scattering in the cluster.^42^ Nahon, Signorell, and their coworkers have employed a coincidence imaging spectrometer at SOLEIL to measure photoemission anisotropy as a function of electron kinetic energy for neutral water clusters up to *n* = 20.^43^ Their results are reproduced in Fig. 7. The anisotropy parameter diminishes in the range *n* = 1–6, while the value becomes almost invariant with *n* up to 20. The result indicated that the formation of hydrogen bonding network changes the character of ionized orbitals, especially 1*b*_1_ and 3*a*_1_ involved in hydrogen bonding, in small clusters. They have also predicted photoemission anisotropy of liquid water using the values of water clusters (*n* = 1 or 6) and Monte-Carlo simulations of the elastic and inelastic scattering of an electron in liquid water.^43^ The green diamonds shown in Fig. 7 are *β*_2_ values determined for water molecules, which almost agree with their experimental values,^42^ although our results are slightly smaller. The *β*_2_ values determined for water molecules in the liquid water spectrum are indicated by blue diamonds. The experimental *β*_2_ values for liquid water indicated by red diamonds are generally in reasonable agreement with the simulation using the water hexamer value, except that an ambiguity remains in our anisotropy parameters owing to possible space charge effects discussed above.

## IV. CONCLUSION

We have constructed an EUV light source using SiC/Mg coated mirrors, which provided a constant photon flux for different polarizations. The performance of our instrument combining the EUV laser and a linear TOF photoelectron spectrometer was verified using photoemission spectroscopy of rare gases and water molecules. We measured the photoemission spectra of liquid water for P and S polarizations without an electric field gradient around a microjet by adjusting the electrolyte concentration. Careful analysis presented in this study illustrated various factors to be considered in the accurate determination of photoemission anisotropy for liquids. The photoemission anisotropy parameters of liquid water were considerably smaller than those of water molecules owing to inelastic scattering in the liquid, and they are in quantitative agreement with the theoretical prediction based on Monte-Carlo simulations.
